# Microbial production and functional assessment of *γ*-polyglutamic acid isolated from *Bacillus* sp. M-E6

**DOI:** 10.3389/fmicb.2025.1647287

**Published:** 2025-10-15

**Authors:** Verma Manika, Palanisamy Bruntha Devi, Jessica Majaw, Potunuru Uma Rani, G. Bhanuprakash Reddy, Digambar Kavitake, Prathapkumar Halady Shetty

**Affiliations:** ^1^Department of Food Science and Technology, Pondicherry University, Pondicherry, India; ^2^Biochemistry Division, ICMR-National Institute of Nutrition, Hyderabad, Telangana, India

**Keywords:** *Bacillus* sp. M-E6, *γ*-polyglutamic acid, ion-exchange chromatography, physico-chemical properties, cryoprotection, flow cytometry

## Abstract

This study describes the production and functional evaluation of *γ*-polyglutamic acid (*γ*-PGA) synthesized by *Bacillus* sp. M-E6, isolated from a traditional fermented food. Among 103 isolates screened, strain *Bacillus* sp. M-E6 exhibited the highest *γ*-PGA yield (4.9 g/L) and was taxonomically identified through 16S rRNA gene sequencing. The *γ*-PGA was extracted and characterized for the physicochemical and techno-functional properties. The *γ*-PGA was purified using ion-exchange column chromatography, and its molecular mass was determined by Electrospray Ionization-Mass Spectrometry (ESI-MS), which found it to be 60.42 kDa. Fourier-transform infrared spectroscopy and nuclear magnetic resonance analysis confirmed the polymer’s structure as *γ*-PGA. Thermogravimetric analysis revealed high thermal stability (>600 °C), while scanning electron microscopy showed a porous and granular morphology. The *γ*-PGA showed potent functional properties such as water holding capacity (196.21%), water solubility index (96.64%), water contact angle (26°), oil binding capacity (104.78%), flocculation activity (29.32%), emulsifying activity (79.33%), and antioxidant activity. Further, the *γ*-PGA showed an excellent cryoprotectant activity with probiotic bacteria *Lacticaseibacillus rhamnosus* (97.57%), *Streptococcus thermophilus* (93.47%), and *Limosilactobacillus fermentum* (84.53%) as evaluated by flow cytometric analysis and direct plate count. These findings highlight the multifunctional potential of *γ*-PGA from *Bacillus* sp. M-E6 as a natural, biocompatible cryoprotectant with promising applications in the food, cosmetic, and biomedical industries.

## Graphical abstract

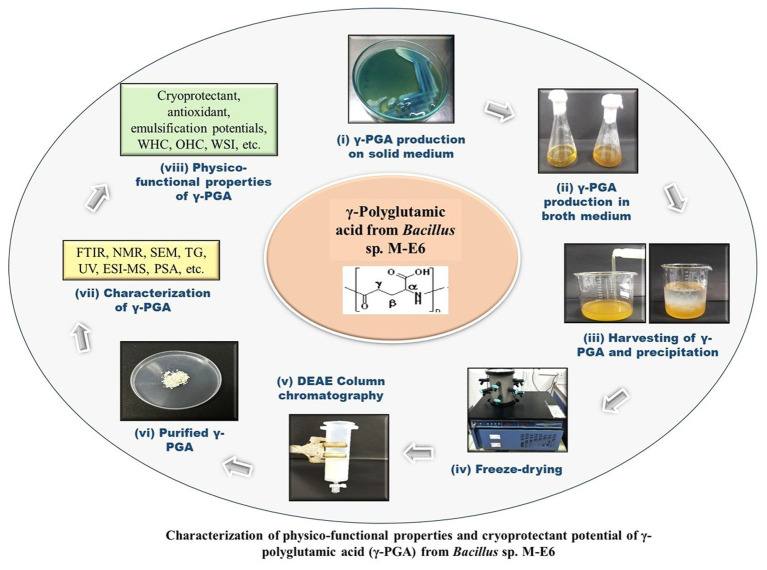

## 1 Introduction

Poly-*γ*-glutamic acid or *γ*-PGA is a palatable, anionic, water-soluble, and biodegradable microbial biopolymer produced primarily by *Bacillus* species. It consists of D-glutamic acid, L-glutamic acid, or a mixture of both enantiomers linked with amide bonds between amino and carboxyl groups (Lee et al., 2020). The *γ*-PGA is known to have Generally Recognized as Safe (GRAS) status and, thus, is considered to be safe (GRAS Notices Inventory, GRN No. 339, U.S. Food and Drug Administration, 2010). Therefore, it is widely used as a versatile additive in food and feed industries, as well as in cryopreservation and drug delivery (Park et al., 2005). Its diverse biochemical properties, influenced by factors such as enantiomeric composition and molecular mass, make *γ*-PGA applicable across a range of fields (Lee et al., 2018). There are two isoforms, i.e., poly-*γ*-glutamic acid (*γ*-PGA) and poly-*α-*glutamic acid (*α*-PGA), distinguished by the position of the attached carboxyl group. Since it is difficult to produce *α*-PGA naturally by the microorganisms, it is synthesized chemically, and the chemical synthesis yields *α*-PGA having a molecular weight lower than 10 kDa, limiting its applications (Buescher and Margaritis, 2007). In contrast, *γ*-PGA is produced by microorganisms with molecular mass ranging between 10 and 10,000 kDa, offering diverse functional properties and potential industrial applications (Ashiuchi, 2013). *γ*-PGA features amide bonds linking *α*-amine group with the *γ*-carboxyl group, distinguishing it structurally from other proteins (Jose Anju et al., 2018). Moreover, *γ*-PGA synthesis occurs in a ribosome-independent manner, where polymerization is facilitated by the enzyme systems located on the membrane.

The *γ*-PGA is mainly produced by the *Bacillus* spp. especially *B. subtilis, B. anthracis, B. thuringensis B. licheniformis, B. cereus, B. amyloliquefaciens, B. pumilus, B. mojavensis, B. atrophaeus, B. megaterium, Lysinibacillus sphaericus, Fusobacterium nucleatum,* and an archaebacteria, *Natrialba aegyptiaca* (Yu et al., 2018). The presence of attached peptidoglycan and the organism producing the *γ*-PGA determine its function. Peptidoglycan can adhere to *γ*-PGA, aiding in the virulence development and functioning as a glutamate source during early stages of starvation. Thus, *γ*-PGA helps bacteria survive starvation, acting as a glutamate source during nutrient deprivation in the late stationary phase.

The viability of most probiotics is reduced upon freezing at extremely low temperatures and during freeze–thaw cycles. However, this can be overcome through the application of cryoprotectant additives. Due to the potential health hazards linked to existing cryoprotectants such as dimethyl sulphoxide (DMSO) and glycerol, there is a demand for cryoprotectants characterized by enhanced safety profiles. The high anionic and acidic amino acid composition of *γ*-PGA contributes to cryoprotectant activity (Shih et al., 2003). Additionally, *γ*-PGA was reported for the cryoprotection of the potential probiotic bacteria such as *Lactobacillus paracasei*, *Bifidobacterium breve, and B. longum* (Bhat et al., 2013). The application of *γ*-PGA demonstrated protective effects on bacterial cells in fruit juices and enhanced their survival under severe conditions of the digestive tract (Jose Anju et al., 2018). Also, it is reported to resist the probiotic bacterium *Bifidobacterium bifidum* during transit in simulated gastrointestinal fluid (Huang et al., 2023). Recently, *γ*-PGA was also reported for its encapsulation potential with probiotics that positively altered gut microbiota and short-chain fatty acid content by maintaining cell viability in the gastrointestinal tract (Jang et al., 2024). The current study describes the extraction, purification, and characterization of a *γ*-PGA produced by *Bacillus* sp. M-E6 isolated from fermented legumes and evaluation of physicochemical and functional properties, including cryoprotectant potential. In addition, explores the understanding of γ-PGA’s cryoprotective spectrum across probiotic species, validated by a high-end approach of flow-cytometry over the conventional plate count method.

## 2 Materials and methods

### 2.1 Materials and chemicals

Diethylaminoethyl cellulose (DEAE cellulose) resin, propidium iodide (PI), brilliant green, dialysis membrane-135 (LA-398), and other chemicals, reagents, and media were procured from Hi-Media Laboratories, India. Potassium bromide (KBr) and quercetin were obtained from Sigma-Aldrich chemicals Pvt. Ltd. (Bangalore, India). Technical grade standard r-PGA was procured from SRL, India. Probiotic cultures *Limosilactobacillus fermentum* (MTCC-9748) and *Lacticaseibacillus rhamnosus* (MTCC-1423) were purchased from Microbial Type Culture Collection (MTCC), CSIR-Institute of Microbial Technology (IMTECH), Chandigarh, India. *Streptococcus thermophilus* (NCDC74) was obtained from National Collection of Dairy Cultures, NDRI, Karnal, India in the form of freeze-dried culture (Haryana, India). Obtained strains were stored at −30 C for further use. Vegetable oils were purchased from the local market. Analytical grade chemicals, solvents, and reagents were used in this study.

### 2.2 Isolation and screening of potential *γ*-PGA producers

Indian indigenous fermented foods such as Idli batter (14 samples- sourced from households, restaurants, street shops, and canteens in and around the Pondicherry University, Puducherry, India), fermented legumes such as black-eyed beans, horse gram, green gram, white chickpea, black chickpea, and fermented dry fish were used for the isolation of *γ*-PGA producing bacterial isolates. Samples were collected, taken to the lab, and processed for the microbiological evaluation. Five grams of each sample were taken, serially diluted using 1x PBS (phosphate-buffered saline, pH 7.2), plated on nutrient agar, and incubated aerobically at 37°C for 24 h. Morphologically different colonies, based on their sliminess, were selected for the preliminary screening of *γ*-PGA production. Selected isolates were streaked on the *γ*-PGA production selective medium consisting of 0.004% methylene blue dye, 1% glucose, 0.05% Potassium di-hydrogen phosphate (KH_2_PO_4_), 0.01% magnesium sulphate heptahydrate (MgSO_4_.7H_2_O), 0.5% monosodium glutamate (MSG), 0.5% yeast extract, 0.05% di-Potassium hydrogen phosphate (K_2_HPO_4_), and 1.5% agar with pH 6.5 ± 0.1 and incubated at 37°C for 24 h. The potent *γ*-PGA producers were confirmed based on the concentric zone formed around the bacterial colonies (Chatterjee et al., 2018).

### 2.3 Production of γ-PGA

The *γ*-PGA positive isolates were grown in 100 mL of the same *γ*-PGA production broth, as mentioned above, in a 500 mL Borosil Erlenmeyer flask without adding the methylene blue dye and incubated aerobically at 37°C under shaking at 200 rpm for 72 h (Peng et al., 2015). The production broth was harvested, and biomass was removed by centrifugation at 13000 × g for 20 min under 4 C. Subsequently, three volumes of absolute pre-chilled ethanol were added to the supernatant and kept overnight for precipitation at 4°C. The precipitated *γ*-PGA was centrifuged and dialyzed against deionized water to remove the low molecular mass impurities and salts. Dialyzed content was freeze-dried for 18 h at −45 C using a lyophilizer (IIShin BioBase model no. TFD5503, South Korea) and used for further analysis.

### 2.4 Confirmation of γ-PGA production

#### 2.4.1 Qualitative analysis

Amino-acid analysis was performed to ascertain the *γ*-PGA producers as per the method reported by Mahaboob Ali et al. (2020) with some modifications. Freeze-dried *γ-*PGA was hydrolyzed in 6 M HCl at 110°C for 12 h, resuspended in 1 mL deionized water, and filtered (0.4 μm syringe filters). HPTLC analysis was performed using a CAMAG TLC sampler 4 with silica gel-60 plates (Merck, Germany) and a mixed mobile phase consisting of butanol: acetic acid: water (3:1:1 v/v) and 96% ethanol/water (63:37 v/v). Samples (4 μL) were spotted against the standard (D and L-glutamic acid) with a controlled band length of 6 mm using a Hamilton syringe. Plates were sprayed with 0.2% ninhydrin in acetone, heated at 100°C for 5 min, and visualized using a CAMAG TLC Visualizer at 254 and 360 nm with winCATS software.

#### 2.4.2 Quantitative analysis of *γ*-PGA

*γ*-PGA produced by potent isolates was further quantified using a UV–Vis spectrophotometer (Shimadzu, UV-1800, Japan) as per the method of Zeng et al. (2013). A series of PGA concentrations (40–200 μg/mL) were prepared with deionized water, and a standard curve was plotted by scanning at 216 nm. Likewise, *γ*-PGA samples extracted from the isolates were dissolved in deionized water, scanned for absorbance at 216 nm, and quantified using a standard curve (Chatterjee et al., 2018).

### 2.5 Identification and selection of potent γ-PGA producing bacteria

The confirmed highest *γ*-PGA producing isolate M-E6 was identified using 16S rRNA gene sequencing at the NCIM (National Collection of Industrial Microorganisms), CSIR-NCL, Pune, India. Briefly, chromosomal DNA was isolated, followed by amplification of 16S rRNA gene (1,500 bp) (Devi et al., 2023). Amplicons were sequenced by Sanger’s method (ABI 3500xL analyzer), a 1,500 bp fragment of the bacterial 16S rRNA gene was amplified using polymerase chain reaction in a thermal cycler, using the forward primer 704F (5’-AGATTTTCCGACGGCAGGTT-3′), reverse primer 907R (5′-CCGTCAATTCMTTTGAGTTT-3′). The PCR reactions were prepared with PCR buffer, 2 mM MgCl_2_ (Invitrogen, United Kingdom), 0.24 mM of each dNTP (Promega, UK), 20 μM of each primer (MWG Biotech, GmbH), and 1 U Platinum Taq DNA polymerase (Invitrogen, UK). Cycling conditions used were, initial denaturation at 95°C for 3 min, 30 cycles of 94°C for 1 min, 54°C for 1 min, and 72°C for 2 min, followed by a final extension at 65°C for 5 min extended to 8 min for last cycle. Amplicons were purified using Exonuclease I-Shrimp Alkaline Phosphatase (Exo-SAP) (Darby et al., 2005). The raw data was edited using CHROMASLITE v1.5 followed by analysis of sequence homology using NCBI-BLAST (Altschul et al., 1990), and phylogenetic tree was developed using Maximum Likelihood (MEGA11). The 16S sequence was deposited in NCBI to obtain a strain accession number.

### 2.6 Bacterial growth kinetics of selected γ-PGA producing strain

The bacterial growth kinetics of isolate M-E6, along with the *γ*-PGA production, was performed as reported by Hatiboruah et al. (2020) with minor modifications. An overnight grown culture (1% v/v) was inoculated into 50 mL of production medium and incubated at 37°C under rotary shaking at 180 rpm. Samples were taken at 24, 48, 72, and 96 h intervals and tested for CFU (Colony Forming Units) count on Luria Bertani (LB) medium. Simultaneously, *γ*-PGA yield and pH of the medium were also measured.

### 2.7 Purification of γ-PGA produced by M-E6

Ion exchange chromatography was used to purify the dialyzed *γ*-PGA, following the method of Bajestani et al. (2018) with minor modifications. DEAE cellulose 52 resin was activated, packed in 50 mL glass syringe (10 cm length, 3 cm diameter). Resin was equilibrated using 30 mM sodium acetate (pH 3.0) followed by loading 2 mL of *γ*-PGA sample (50 mg/mL). The column was gradually eluted with a gradient concentration of NaCl (0.1, 0.5, 0.75, and 1.0 M), keeping the flow rate at 1 mL/min. Fractions were collected, quantified for *γ*-PGA content, and lyophilized. Phenol sulphuric acid, Lowry’s, and glutamate dehydrogenase-coupling assays were performed to confirm the elimination of polysaccharides, proteins, and glutamate, respectively (Ashiuchi et al., 1999).

### 2.8 Characterization of γ-PGA produced by M-E6

#### 2.8.1 Molecular mass determination

The purified *γ*-PGA was subjected to an electrospray ionization mass spectrometer (ESI-MS) (Agilent Technologies 6,530 Accurate-Mass Q-TOF LC/MS) for its molecular mass determination. The sample was prepared using acetonitrile (0.5 mg/mL), and the spectrum was recorded. Furthermore, an online tool (ESIprot) was used to calculate the charge state and molecular mass of the *γ*-PGA. The molecular mass was determined by selecting the chosen ion peaks from the spectrometric scan (Winkler, 2010).

#### 2.8.2 Fourier transform infrared spectra (FTIR) analysis

FT-IR spectroscopy (Thermo Nicolet 6,700, USA) analysis was performed with a spectrum wavelength range from 4,000–400 cm^−1^ to examine the structural and functional groups of *γ*-PGA. Briefly, *γ*-PGA sample (5 mg) was mixed with potassium bromide (KBr), squashing the homogenous mixture on a metallic disc into a pellet, and scanning was performed (Lee et al., 2018).

#### 2.8.3 Nuclear magnetic resonance (NMR) spectroscopy

The spectra for both proton (^1^H) and carbon (^13^C) were recorded in a Bruker DRX advance 400 mHz spectrometer. The γ-PGA sample was dissolved in D_2_O with 1% (w/v) concentration using 10 mm NMR tubes. The ^1^H NMR spectra were recorded at a frequency of 400 MHz, and the chemical shifts were expressed in parts per million (ppm), with hydrodeoxygenation at 4.8 ppm as an internal reference. Peak areas in the ^1^H-NMR spectra were quantified through digital integration and are presented as relative peak areas corresponding to a specific number of hydrogens. The chemical shifts in the ^1^H spectra were measured relating to 3-(trimethylsilyl) propionic acid-d4 sodium salt (TSP). The ^13^C NMR spectra were recorded at a frequency of 100 MHz, and the chemical shifts are expressed in parts per million (ppm), presenting the types of carbon in the structure (Ho et al., 2006; Lee et al., 2018).

#### 2.8.4 Thermal gravimetric analysis (TGA)

To study the thermal stability and to evaluate the thermal degradation temperature (T_d_) of *γ*-PGA, thermogravimetric analysis (TGA) was performed on a thermal system (Q600 SDT). TGA was carried out with 10 mg of purified *γ*-PGA in an N_2_ atmosphere at a flow rate of 10 mL/min over a temperature range of 0–600°C with a heating rate of 10 C/min (Lee et al., 2018).

#### 2.8.5 Scanning electron microscopy (SEM)

The morphology of *γ*-PGA was assessed by using scanning electron microscopy (SEM). Powdered *γ*-PGA (2 mg) was coated with carbon using a sputter on an aluminium stub and analyzed under SEM (Hitachi, Model: S-3400 N), followed by image analysis using S-3400 software (Madgulkar et al., 2016).

### 2.9 Physicochemical properties of *γ*-PGA

#### 2.9.1 Water contact angle

For contact angle measurement, 5 mg/mL of *γ*-PGA sample was dispersed in deionized water, followed by vortexing for 5 min. The sample was coated on a glass slide, and subsequently contact angle was measured using an optical tensiometer (Theta lite, Biolin Scientific) equipped with a video-based camera. Results were generated using One Attension version 2.4 software (Benito-Gonzalez et al., 2019).

#### 2.9.2 Particle size distribution

To examine the particle size of *γ*-PGA, a 0.5%, w/v sample was dissolved in deionized water and analyzed by a nanoparticle size analyzer using a Zetasizer Nano ZS (Malvern Instruments Ltd., Great Malvern, UK) at 25°C (Kavitake et al., 2016).

#### 2.9.3 Water holding capacity (WHC)

The protocol of Ye et al. (2018) was followed to estimate the WHC of *γ*-PGA with minor modifications. The *γ*-PGA sample, 2.5 mL (100 mg/mL) was prepared with Milli Q water and mixed using a vortex mixer for 10 min in a pre-weighed centrifuge tube. The resulting solution was subsequently centrifuged at 3000 × g for 40 min. The pellet was reweighed, and WHC was calculated as,


$$\mathrm{WHC}\;(\%)=\frac{\text{Weight of sample bound water}}{\text{Initial weight of sample}}X\;100$$

#### 2.9.4 Water solubility index (WSI)

To determine WSI, 250 mg of powdered *γ*-PGA was taken in 6 mL of deionized water and dissolved properly. Then the solution was vortexed for 20 min and centrifuged at 3000 × g for 10 min. Subsequently, supernatant was dispensed to a Petri dish and kept for drying at 105°C for 8 h, then the dry solid weight was recorded (Ahmed et al., 2013). WSI was calculated as:


$$\mathrm{WSI}=\frac{\mathrm{Dry}\;\text{solid weight in supernatant}}{\text{Initial sample weight}\;}X\;100$$

#### 2.9.5 Oil binding capacity (OBC)

The OBC of *γ*-PGA was assessed following Sun et al. (2022) method with some modifications. Soybean oil (10 mL) was mixed with 200 mg *γ*-PGA in a pre-weighed centrifuge tube. The centrifuge tube was incubated for 30 min and the blend was mixed on a vortex every 10 min interval. Centrifugation was done for 10 min at 10,000 rpm. Subsequently, the supernatant was removed, and the weight of residual content in the tube was recorded. The OBC was calculated based on the following formula:


$$\mathrm{OBC}\;(\%)=\frac{\text{Oil bound weight}}{\text{Initial weight}\;}X\;100$$

#### 2.9.6 Emulsifying activity (EA) and stability (ES)

Emulsifying activity of *γ*-PGA was investigated as per the method outlined by Ashtaputre and Shah (1995) and Kavitake et al. (2024) with slight changes. Vegetable oils such as olive (Gaia, extra virgin), sunflower (Organic nation, cold pressed) and coconut oil (cold pressed) were utilized to estimate the emulsifying potential of *γ*-PGA. Three mL aqueous *γ*-PGA solution (10 mg/mL) was mixed in 3 mL of the respective oil and vigorously agitated for 2 min on a vortex. The emulsions were observed for emulsifying activity after an hour, and stability was calculated after 24 h. The aqueous and emulsion layers were measured and used to calculate the emulsifying activity (EA) and stability (ES) using the formula:


$$\mathrm{EA}/\mathrm{ES}=\frac{\text{Volume of the emulsion layer}}{\text{Total volume}\;}X\;100$$

#### 2.9.7 Flocculation activity

The flocculation capacity of *γ*-PGA samples was estimated using the method described by Devi et al. (2016) with minor modifications. Ten milliliters of activated-charcoal carbon suspension (5 g/L) was taken with a subsequent addition of 0.5 mL CaCl_2_ (6.8 mM) and 0.5 mL *γ*-PGA (2 mg/mL), and a control was prepared without adding *γ*-PGA. The solutions were then mixed on a vortex mixer for 30 s and left undisturbed at room temperature for 15 min. The absorbance of the aqueous phase was read at 550 nm using EON Gen 5 BioTek microplate reader. The flocculating activity was determined based on the following equation,


$$\text{Flocculating acitivity}\;(\%)=\frac{\left(\text{Control}-\text{sample}\right)}{\text{Sample}\;}X\;100$$

### 2.10 Antioxidant activity of *γ*-PGA

#### 2.10.1 ABTS (2,2′-Azino-bis-3-ethylbenzothiazoline-6-sulphonic acid) assay

The antioxidant potential of *γ*-PGA was assessed by using the ABTS^+^ radical scavenging assay following the methodology reported by Abdel-Hamid et al. (2019). ABTS^+^ solution (7 mM) was prepared by mixing 88 μL of 140 mM potassium persulfate with 5 mL of ABTS solution, followed by incubation in the dark at room temperature to produce ABTS^+^ radicals (cationic). This was followed by dilution of ABTS^+^ solution with water until the OD reached 0.7 to 0.73 at 734 nm. Then, ABTS^+^ solution was added to different concentrations (2.5–20 mg/mL) of *γ*-PGA solution in the ratio 1:1. The mixture was incubated at room temperature for 6 min, and the absorbance was recorded at 734 nm. Quercetin was kept as a positive control, and the ABTS^+^ scavenging potential of *γ*-PGA was determined based on the following formula:


$$\text{Sacvenging activity}\;(\%)=\frac{\text{Control}-\text{Test}}{\text{Control}\;}X\;100$$

#### 2.10.2 Reducing activity

To assess the reducing activity of *γ*-PGA, various concentrations (2.5–20 mg/mL) of *γ*-PGA solutions were tested following the method described by Das and Goyal (2015). The test sample (200 μL) was added to the reaction combination comprising 200 μL of ferricyanide and 20 mmol/L sodium phosphate buffer (pH 7). This mixture was kept under incubation for 20 min in a water bath at 50°C. Further, an additional 200 μL of trichloroacetic acid (10%) was added to cease the reaction. The solution was centrifuged at 2000 rpm for 10 min at 4°C. Then, the supernatant was added to 0.1% of 100 μL ferric chloride and 400 μL of deionized water. Deionized water was taken as a control for the analysis. Absorbance was read at 700 nm, and the reducing power was demonstrated as μg/mL of ascorbic acid equivalent (AAE).

#### 2.10.3 Hydroxyl radical scavenging

The Fenton reaction was carried out to assess the hydroxyl radical scavenging activity of *γ*-PGA. The reaction mixture includes different concentrations of sample dissolved in deionized water, 1 mL of 0.435 mM brilliant green, 2 mL of 0.5 mM FeSO_4_.7H_2_O and 1.5 mL of 3% (v/v) H_2_O_2_. The reaction mixture was kept under incubation for 20 min at room temperature. Absorbance was read at 624 nm, and ascorbic acid was kept as a positive control (Zhang et al., 2013). Hydroxyl radical scavenging activity is calculated as:


$$\text{Scavenging acitivity}\;(\%)=\frac{\left(\mathrm{As}-\mathrm{Ao}\right)}{(A-\mathrm{Ao})\;}X\;100$$

where, A_s_ is the absorbance of the sample’s reaction, A_o_ is the absorbance of the control reaction mixture without the sample, and A is the absorbance of the reaction mixture with only water.

#### 2.10.4 Ferric-reducing antioxidant power (FRAP)

Ferric-reducing antioxidant power of *γ*-PGA was investigated at different concentrations (0.25–20 mg/mL). FRAP reagent preparation was done by mixing 0.3 M acetate buffer (pH 3.6), 0.01 M 2,4,6-tris(2-pyridyl)-s-triazine (TPTZ) in 0.04 M HCl, and 0.02 M FeCl_3_ in the ratio 10:1:1. To 2.7 mL of freshly prepared FRAP reagent, 300 μL of sample solution was added. The whole mixture was kept in an incubator at 37°C for 10 min, and the absorbance was recorded at 593 nm. A calibration curve was plotted using ferrous sulfate, and the result was demonstrated as micromoles of ferrous equivalents per mg of *γ*-PGA (μmol Fe(II)/mL). Ascorbic acid was kept as a positive control, and water as a negative control (Bomfim et al., 2020).

### 2.11 Cryoprotectant potential of γ-PGA

#### 2.11.1 Standard plate count method

Cryoprotection assays were performed as per the method reported by Bhat et al. (2013) with some minor adaptations. Briefly, purified *γ*-PGA was autoclaved to sterilize at 110°C and 0.35 bar for 30 min. Cells of *Lmb. fermentum, LcS. rhamnosus* and *S. thermophilus* were cultured anaerobically in 100 mL each of MRS (de Man, Rogosa, Sharpe) broth for 22 h followed by viable cell counting before freeze–thawing. The cultures were centrifuged to get the cell pellet followed by cell washing with 1x PBS and then resuspended in 10 mL solutions of PBS with respective cryoprotectants such as 5% (w/v) *γ*-PGA, 10% (w/v) *γ*-PGA corresponding to 10% (w/v) sucrose and 10% (v/v) glycerol as positive controls and sterile distilled water without any cryoprotectant was taken as negative control. The suspensions were incubated at room temperature for 1 h and at −80°C in a deep freezer (Thermo Fisher Scientific 902GP) for 1 h, followed by thawing at room temperature for 20 min, repeating the process 4 times. Cell suspensions were then incubated at −80°C for 24 h, and cell viability was determined. Enumeration was performed using the Miles and Misra technique, a 10-fold dilution series followed by plating of each cell suspension in hexaplet, then incubated at 37°C (Miles et al., 1938). Additionally, this was subjected to flow cytometric analysis for the enumeration of cell viability.

#### 2.11.2 Fluorescence staining and flow cytometry data acquisition

In addition to the standard plate count, a rapid cytometric analysis for non-viable/non-culturable cells was performed using an effective fluorescent probe, Propidium Iodide (PI) with fluorochrome phycoerythrin (PE) (Foglia et al., 2020), and distinguished from live/viable cells in a mixed population. The fluorescent dye propidium iodide (PI) stains the cell nuclear material by entering the damaged cell membrane. Dead cells with compromised membranes get stained with PI, while live cells with intact membranes are impermeable to PI. PI concentration (41 nM) was optimized for all three strains of *LcS. rhamnosus, Lmb. Fermentum,* and *S. thermophilus* (Michelutti et al., 2020). Cells protected with glycerol and sucrose were used as positive controls, and cells suspended in distilled water were used as negative controls.

Following treatment with various cryoprotectants, cells were diluted in sterile phosphate-buffered saline solution up to E-05 dilution, stained with PI, and incubated at room temperature for 15 min. Unstained bacterial cells were used to set the noise signal and threshold. The initial settings in CytoFLEX were followed as per CytoFLEX set up guide (B53767). The flow-cytometer settings optimized for acquisition were: Threshold-FSC 50,000 arbitrary units (a.u.); SSC-10,000 a.u. and Gain at 1000 a.u.; logarithmic amplification. While for PE (Red fluorescence detector) gain was set at 300 a.u. A physical gating of FSC vs. SSC plot was designed to identify the bacterial population. Automatic compensation was applied by importing CytExpert compensation matrix (Acquired with compensation beads stained with fluorochromes PE vs. FITC) from CytExpert software. For single PI (PE) stained cells gating was done in the SSC-A vs. PE-A plot as well as PE vs. FITC to identify the single stained dead cells. Prior to acquisition of single stained dead cells, PE and FITC stained compensation beads were used to set the compensation matrix. Flow acquisition rates were kept at 1000 events/s and a total of 10,000 events per sample were acquired using Beckman coulter CytoFLEX. Gating for single stained cells with PI (PE) was done for almost 90 to 96% of acquired events. Assays were performed in hexaplets and data was analyzed using CytExpert software version 2.0. Results were expressed as the percentage of live/culturable and dead/non-culturable cells (Deza et al., 2017).

### 2.12 Statistical analysis

All the analysis were conducted in triplicate, and the results were expressed as mean ± SD. The results were statistically analyzed using Microsoft Excel 2013 and IBM SPSS Statistics software (version 20). One-way ANOVA test (Duncan’s test) was applied to compare the data and find out the significant difference (*p* < 0.05).

## 3 Results and discussion

### 3.1 Screening of *γ*-PGA producing bacteria

A total of 103 bacterial isolates from various fermented foods were screened for *γ*-PGA production, and 16 were found to be potential *γ*-PGA producers (Table 1). Positive isolates produced a thick, sticky, mucous-like layer around them on the solid media (Fig. S1A). This phenomenon occurs because of *γ*-PGA, being a polyanionic biopolymer composed of glutamic acid residues, carries multiple negatively charged carboxyl groups. These negatively charged sites readily interact with the cationic nature of basic dyes such as methylene blue, especially in neutral to mildly acidic aqueous conditions. The binding of methylene blue to *γ*-PGA alters the local distribution of the dye, thereby producing a visible halo or zone that serves as an indirect indicator of *γ*-PGA production by the bacteria (Khalil et al., 2017; Chatterjee et al., 2018).

**Table 1:** Presumptive *γ*-PGA-producing isolates per sample tested.

Sample	Number of isolates	*γ*-PGA producers
Idli-batter	78	3
Black-eyed beans	8	3
Horse-gram	3	2
Green-gram	3	3
White chickpea	8	3
Black chickpea	2	2
Dried fish	1	-

### 3.2 Quantification of *γ*-PGA

The selected 16 isolates were inoculated in *γ*-PGA production broth for 72 h and quantified spectrophotometrically. Studies reported that *γ*-PGA displays the maximum absorbance at 216 nm, a rapid method for its quantification (Zeng et al., 2012; Zeng et al., 2013). The yield of *γ*-PGA for 16 isolates ranged from 0.85 to 4.9 g/L (Table 2), where isolate G3 produced the lowest and M-E6 the highest. By HPTLC analysis, all the isolates showed a band corresponding to the standard L-glutamic acid with an R_f_ value of 0.34, thus confirming the product comprising L-glutamic acid units of *γ*-PGA (Fig. S2). Similar results have been obtained by the study performed by Thapa et al. (2021) on the *γ*-PGA extracted from kinema (a traditional soy bean fermented food originated in Eastern Himalayas, specifically in Nepal, Sikkim and Darjeeling) water, and soil samples. Isolate M-E6 was selected for further studies based on band intensity on the TLC plate and the highest yield by spectrophotometric quantification.

**Table 2:** The quantity of *γ*-PGA produced by food isolates using UV-spectroscopic method.

Name of the isolate	PGA produced (g/L)
M-E6	4.9
M-4	4.49
F1	3.82
M-J7	3.46
M-SWR	3.28
HG1	2.88
E2	2.4
GG2	2.1
C5	2.09
GG1	2.03
E3	1.74
Y3	1.32
Ibj6	0.85
G4	0.84
HG2	0.83
G3	0.82

### 3.3 Identification of *γ*-PGA producer

16S rRNA sequencing and molecular phylogeny analysis were used to characterize the potent *γ*-PGA producing isolate M-E6. MEGA11 software was used to construct a phylogenetic tree by the maximum likelihood method (Fig. S3). Isolate M-E6 was identified as *Bacillus* sp. M-E6, and the sequence accession number PV555073 was acquired from the NCBI database. So far, *Bacillus* spp. are the ones that fall under the category of highest *γ*-PGA producers from different fermented foods like *Chungkookjang* (Park et al., 2005), *Sago* (Mohanraj et al., 2019), *Kinema* (Thapa et al., 2021), and *Natto* (Ma et al., 2024).

### 3.4 Bacterial growth and *γ*-PGA production

**Figure 1:**
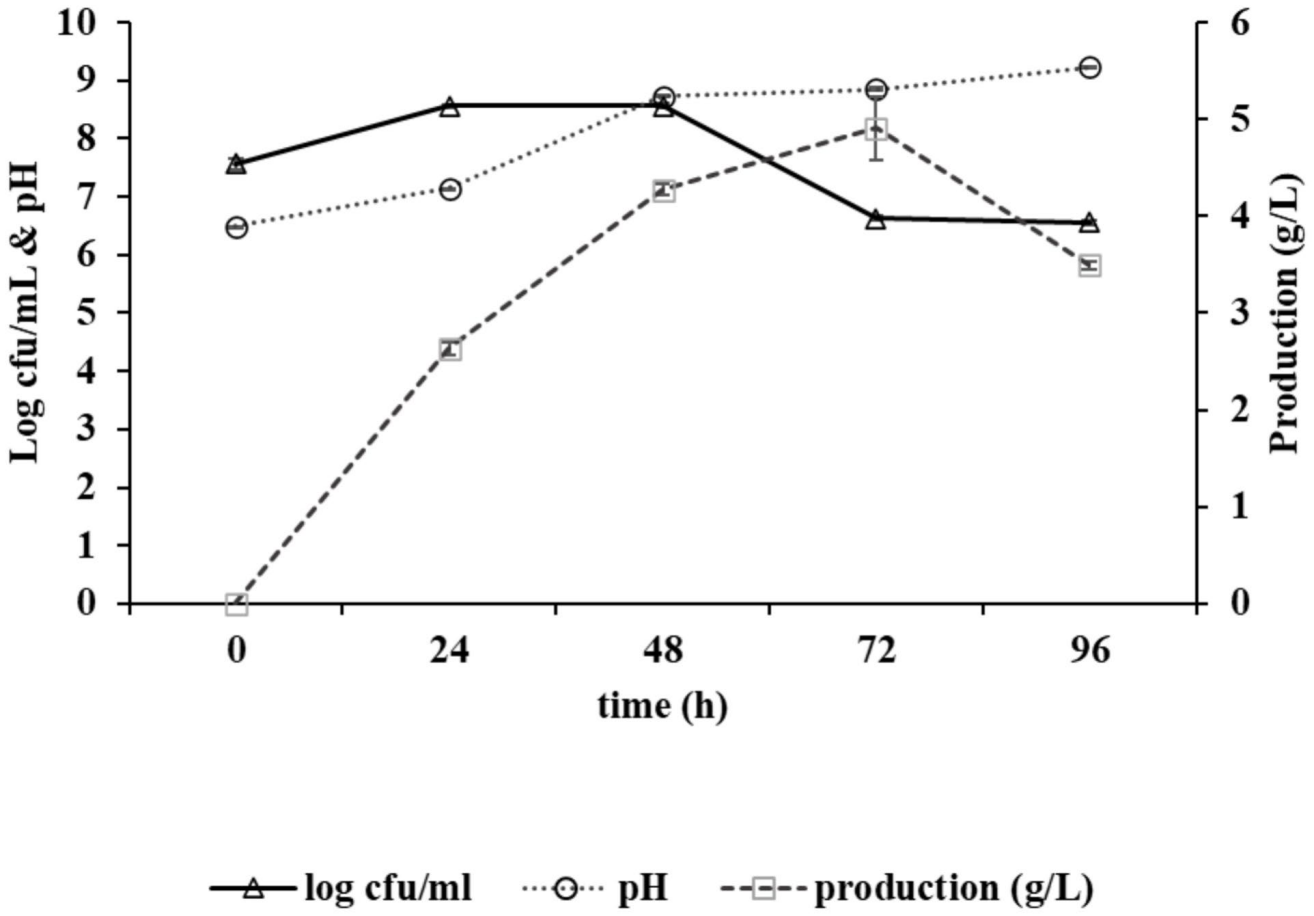
Graph depicting growth kinetics of *Bacillus* sp. M-E6 strain during *γ*-PGA production and changes in pH.

The growth curve of the strain *Bacillus* sp. M-E6 appeared as a sigmoid shape consisting of a lag phase of nearly 6 h, followed by an exponential phase up to 48 h, and the stationary phase up to 72 h. The long exposure of exponential to stationary phase of *Bacillus* sp. M-E6 may be one of the reasons it results in the highest *γ*-PGA yield, as it is evident from the earlier report that the maximum production was achieved during late exponential to stationary phase (Richard and Margaritis, 2003). The rate of production showed a linear pattern, starting afterthe exponential phase, increasing gradually from 2.6 to 4.9 g/L, achieving the maximum yield during 24–72 h while moving toward the late exponential and stationary phase (Figure 1). These observations are in agreement with the earlier study reported by Meissner et al. (2015). An extended incubation resulted in the reduction of *γ*-PGA production, probably due to the utilization of free glutamic acid once the readily available nutrient in the medium is utilized entirely. An increase in sliminess and viscosity of the production medium was observed with an increase in incubation time. Similar findings of *γ*-PGA production in the stationary stage have been reported by Ogawa et al. (1997), Kimura et al. (2004), and Chen et al. (2010). The pH of medium was also noted during *γ*-PGA production, and it was found that there was a linear rise in pH from 6.4 to 9.2 up to 72 h, and it remained stable for another 24 h (Figure 1). It is evident from the reports that *γ*-PGA is effectively produced at neutral or near neutral pH (Bajaj and Singhal, 2011).

### 3.5 Purification of *γ*-PGA

**Figure 2:**
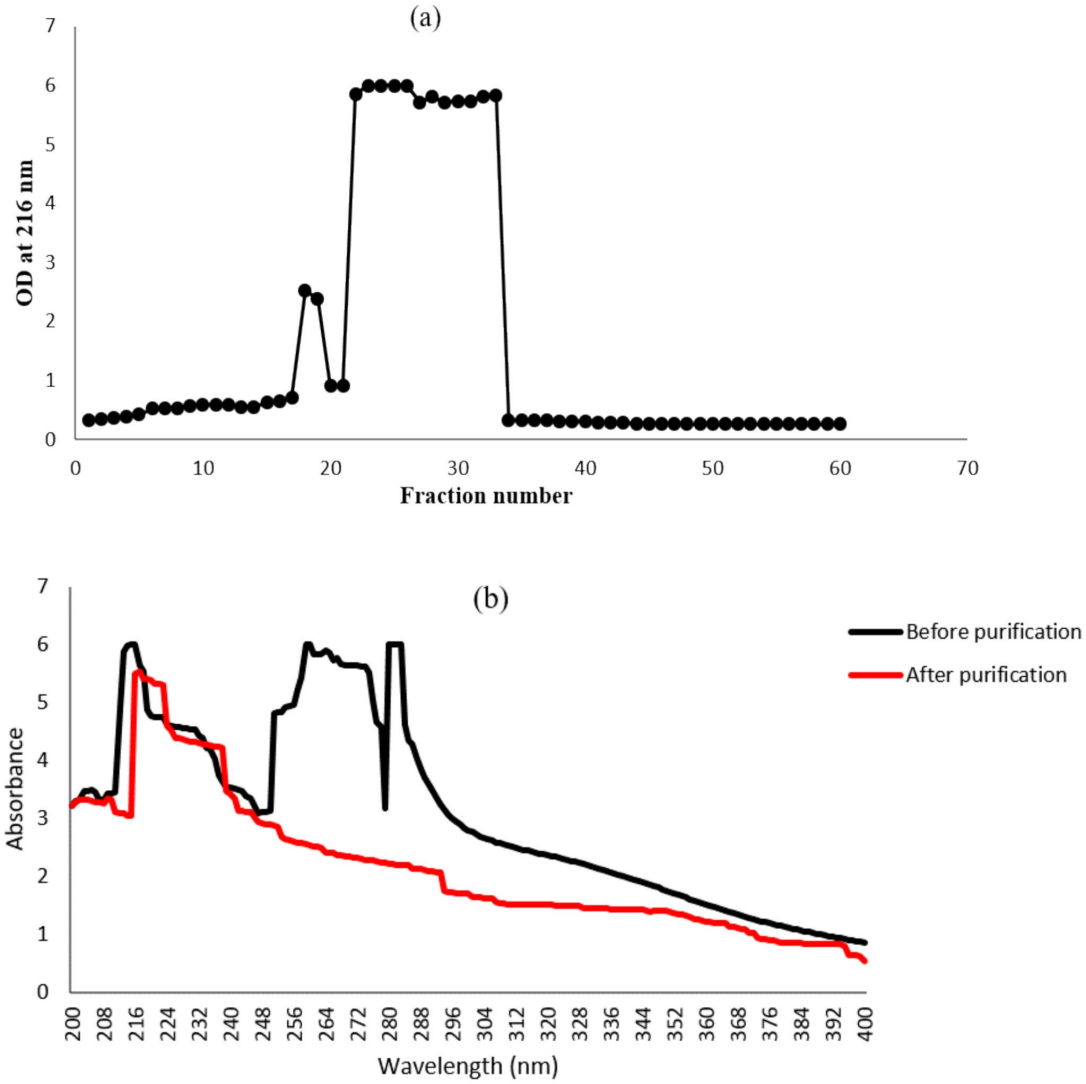
Anion-exchange column chromatography for *γ*-PGA purification using DEAE cellulose-52 resins. **(a)** Fractions eluted with NaCl gradient solution (0, 0.1, 0.5 and 1 M). **(b)** UV spectra of major *γ*-PGA fraction before and after column purification.

Purification of *γ*-PGA is a straightforward approach consisting of three major steps: separation of bacterial biomass by centrifugation, precipitation of *γ*-PGA using ice-cold absolute ethanol, and dialysis of the crude sample to remove low-molecular-mass impurities (Goto and Kunioka, 1992; Tork et al., 2015). In anion-exchange chromatography, the major peak (fraction no. 28–34) was eluted with 0.5 N NaCl (Figure 2a), which marked the presence of *γ*-PGA as revealed by the TLC and UV-spectroscopy (at 216 nm). No peak was detected at 260 and 280 nm (Figure 2b), corroborating the absence of protein and nucleic acid impurities from the *γ*-PGA, which has an absorption peak at 216 nm.

### 3.6 Molecular mass determination

**Figure 3:**
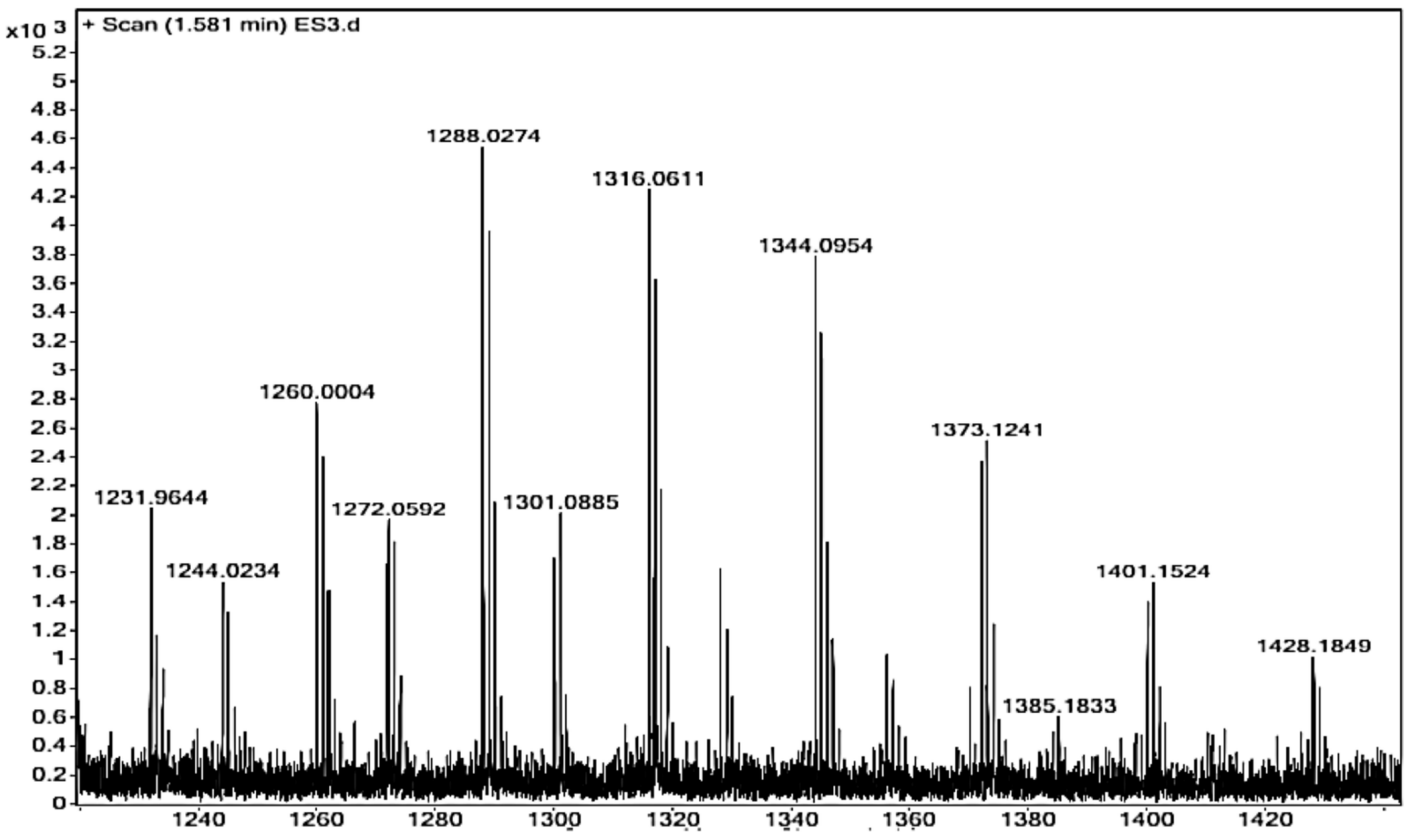
ESI-MS spectrum of purified *γ*-PGA for molecular mass determination.

The molecular mass of the purified *γ*-PGA from the strain *Bacillus* sp. M-E6 was calculated using ESIprot, an open-source online tool based on an ESI-MS spectrum (Figure 3), and was found to be 60.42 kDa with an absolute deviation of only 0.06 kDa. Similarly, Kongklom et al. (2017) found the *γ*-PGA of around the same molecular mass (63 kDa) range from *Bacillus licheniformis* under fed-batch fermentation. According to Li et al. (2022) and Xiong et al. (2012), *γ*-PGA with a molecular mass in the range of 45–66 kDa demonstrates significant potential for applications in drug delivery systems and tissue engineering nanocomposites. A study reported by Guo et al., 2025 presents *γ*-PGA of molecular weight 1,390 kDa, produced by *B. velezensis* isolated from rhizosphere soil. *γ*-PGA isolated from Chinese traditional *Douchi,* strain *B. velezensis* CAU263 by solid-state fermentation reports to have a molecular weight of 3.8 × 10^8^ Da, significantly improved specific volume of bread and thus reduced hardness (Liu et al., 2022). There are reports of high and ultra-high molecular weight *γ*-PGA produced by *B. subtilis* SJ-10 (Lee et al., 2014a, 2014b) and *B. licheniformis* NRC20 as 400 and 1,266 kDa (Tork et al., 2015), respectively, possessing applications in medicine and nanotechnology.

### 3.7 FT-IR spectroscopy

**Figure 4:**
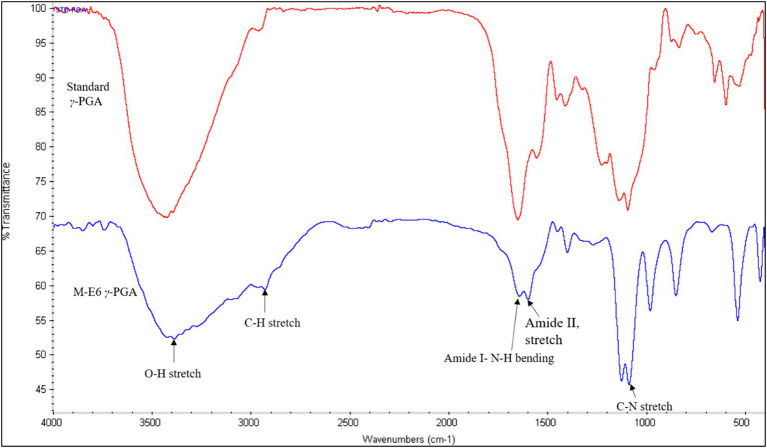
FTIR-spectrum of standard *γ*-PGA (red) and *Bacillus* sp. M-E6 *γ*-PGA (blue).

The presence of four characteristic *γ*-PGA peaks appeared at 3425 cm^−1^, 1,650 cm^−1^, 1,556 cm^-1,^ and 1,094 cm^−1^ corresponding to OH-stretch, amide I (NHbending band), amide II (stretching band), and C=O symmetric stretching band, respectively (Figure 4). The presence of O-H (hydroxyl group) confirms a compound as a polymer. Additionally, a broad peak observed between 2,800–3,000 cm^−1^ overlaps O-H, C-H, and N-H stretching vibrations. The presence of a small peak at 2949 cm^−1^ shows the C-H stretching vibrations. The absence of a strong peak at 1750 cm^−1^ and the presence of the above-mentioned four major peaks confirm the product as *γ*-PGA (Lee et al., 2018; Ho et al., 2006; Pereira et al., 2012; Soliman et al., 2005). Similar findings have been reported by Lee et al. (2018) and Pereira et al. (2012), respectively.

### 3.8 Nuclear magnetic resonance (NMR) analysis of *γ*-PGA

**Figure 5:**
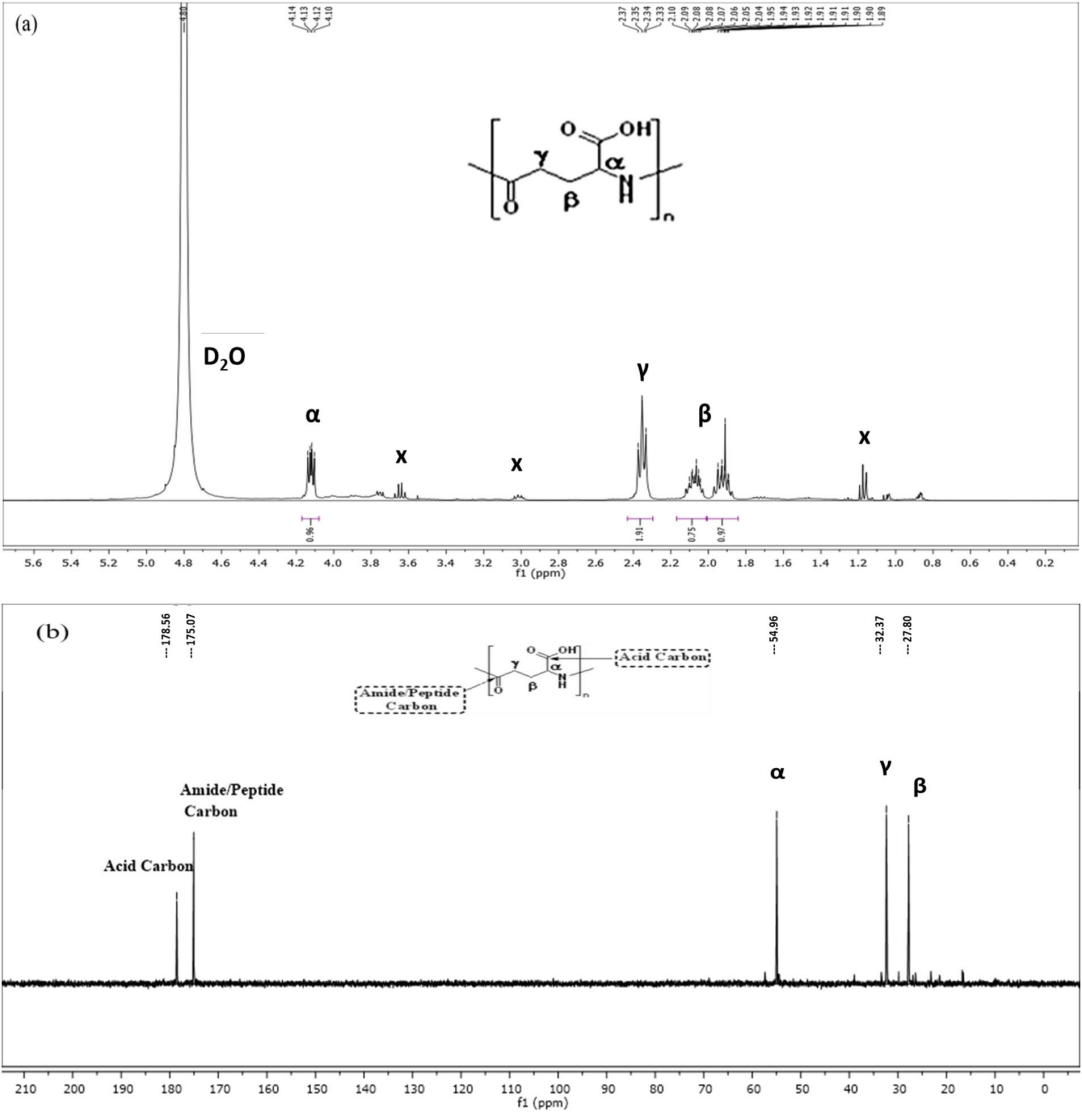
Nuclear magnetic resonance (NMR) spectroscopy of *Bacillus* sp. M-E6 *γ*-PGA **(a)** ^1^H NMR **(b)** ^13^C NMR.

Proton and carbon (^1^H and ^13^C) NMR were carried out to analyze the structure of the *γ*-PGA. The proton spectra for *γ*-PGA in D_2_O reveals chemical shifts for the *α*-CH proton (4.12 ppm), *γ*-CH_2_ proton (2.35 ppm), and *β*-CH_2_ proton (2.07 and 1.92 ppm) (Figure 5a). Furthermore, the ^13^C NMR spectra revealed chemical shifts at 54.96 ppm, 32.37 ppm, 27.80 ppm, 175.07 ppm, and 178.55 ppm correspond to the *α*-CH_2_, *γ*-CH_2,_ and *β*-CH_2_ group, amide carbonyl group (CONH), and COOH group, respectively, of *γ*-PGA (Figure 5b). These NMR results are in line with the findings of Ho et al. (2006), affirming the presence of *γ*-PGA.

### 3.9 Thermogravimetric analysis (TGA)

**Figure 6:**
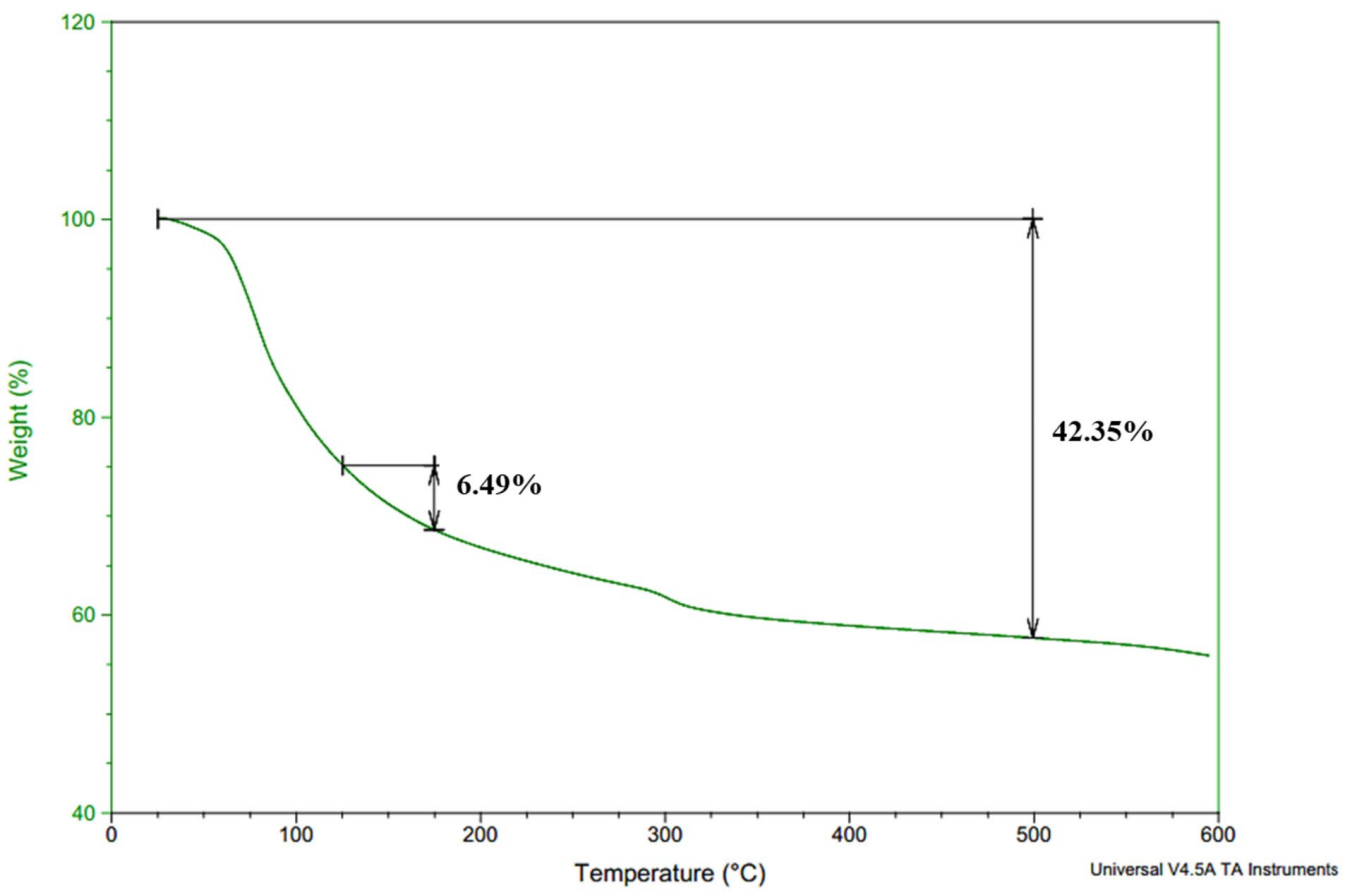
Thermal decomposition temperature curve of *Bacillus* sp. M-E6 *γ*-PGA.

TGA was performed to determine the thermal stability of *γ*-PGA, which is represented by the thermal decomposition temperature (T_d_) of the compound. The T_d_ for the *γ*-PGA was above 600°C (Figure 6), and the *γ*-PGA was found to be stable between 125 and 175°C with a weight loss of just 7%. The sample showed only 42% weight loss at 500°C. However, the degradation temperatures of *γ*-PGA reported earlier were 250°C from *Bacillus subtilis* BL53 (De Cesaro et al., 2014), 250–300°C and 223°C from *Bacillus subtilis* (natto) (Ho et al., 2006). The drastic decrease in weight loss percentage from 200 to 500°C is likely attributable to cyclodepolymerization of the polyacid chain as heat converts the glutamic acid residues into its cyclized form (pyro-glutamic acid). Therefore, it can be deduced that longer the *γ*-PGA chain higher will be its T_d_ as the extended polymeric structure delays the time required for cyclodepolymerization to degrade half the chain (Portilla-Arias et al., 2007).

### 3.10 Scanning electron microscopy (SEM)

**Figure 7:**
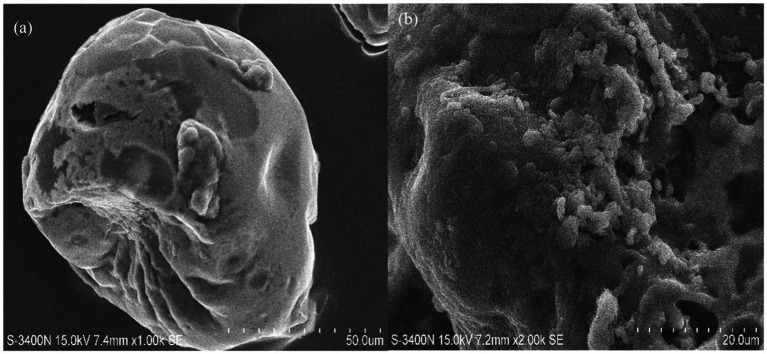
Scanning electron microscopy images of *Bacillus* sp. M-E6 *γ*-PGA at different magnifications: **(a)** 1,000 x **(b)** 2,000 x.

SEM images of the *γ*-PGA polymer at 1000x displays a clear globular structure having both rough and smooth surfaces (Figure 7a). At higher magnification (2000x) *γ*-PGA gave a detailed microstructure showing thin porous layers composed of sponge like granules (Figure 7b). Due to its porosity in the structure, it can be well utilized as food stabilizers, thickening agents in foods and it is also a good choice for the preparation of hydrogels and could be used for the delivery of bioactive compounds, drugs, and probiotics (Khan et al., 2007). A recent study reported by Zhang et al. (2025) proved to improve viscoelasticity and gelling in low-fat yogurt due to enhanced crosslinking between *γ*-PGA and casein micelles, owing to the porous microstructure of *γ*-PGA.

### 3.11 Physico-chemical properties of γ-PGA

#### 3.11.1 Contact angle and particle size distribution

Water contact angle measurements were performed to assess the affinity of *γ*-PGA with water. The change in contact angle of *γ*-PGA was observed over 10 s and found to decrease in a linear manner, reflecting the extremely hydrophilic nature of each sample. The water contact angle of *γ*-PGA was recorded as 26°C, which supports the good water binding ability of *γ*-PGA (Ho et al., 2006; Benito-Gonzalez et al., 2019). The particle size distribution of the *γ*-PGA sample was determined based on the peak intensity shown in Fig. S4. The average hydrodynamic diameter size of this *γ*-PGA molecule was found as 396.5 nm. The polydispersity index (PI) was calculated as 0.440.

#### 3.11.2 Water holding capacity (WHC) and water solubility index (WSI)

WHC of the powdered *γ*-PGA sample was 196.21%, and WSI was 96.64%. The porous matrix structure of *γ*-PGA could have contributed to high water solubility index and holding capacity due to the presence of hydrogen bonds, making it absorptive in nature by holding huge quantities of water (Maina et al., 2008; Feng et al., 2018). It has been reported by Yang et al. (2015) that water solubility is directly related to chain length, branching, linkage arrangements, and degree of polymerization. The study shows good WHC and WSI, thus can easily pave the way in food industries as stabilizing and water-absorbing agents (Pan and Tang, 2023).

#### 3.11.3 Oil binding capacity (OBC)

The ability of an organic compound to adsorb onto the surface of polypeptides or any other substance is referred to as oil binding capacity (OBC). The OBC of the *γ*-PGA was observed to be 104.78 ± 3.45%. The respectable value of OBC may be due to the porous structure of *γ*-PGA, making oil adsorb through it (Insulkar et al., 2018). The findings suggest that the oil binding capacity of a polymer is influenced not only by its type but also by factors such as its chemical composition, hydrophilicity, porosity, and the oleophile nature of the molecule, which plays the most crucial role (Insulkar et al., 2018).

#### 3.11.4 Emulsifying activity and stability

The emulsifying activity of *γ*-PGA was found to be highest (79.33 ± 1.15%) with olive oil among all the oils used in this study. In addition, activity with coconut and sunflower oil was 45.45 ± 0% and 44.84 ± 1.04%, respectively. The concentration of the oil phase relative to the continuous phase are critical factors that influence emulsion stability. Type of oil, emulsifier concentration, temperature and droplet size are important factors that affect emulsion stability. Small droplets with a narrow size distribution enhance stability while reducing coalescence capacity (Ghasemi et al., 2020). When observed after 24 h, the emulsion was found to be stable with olive oil, with an emulsion stability (ES) of 70%. However, with sunflower and coconut oil, it got reduced to 40 and 34% respectively, making them quite unstable (Table 3). The reduction in stability might have occurred due to saturation of oil in turn, increased coalescence in coconut and sunflower oil (McClements et al., 2017; Tiwari et al., 2025). Similar reports were also found for other microbial metabolites, such as exopolysaccharides by Kavitake et al. (2024). Emulsion stability is an essential factor to consider in various industries such as food and pharmaceuticals, where the formation and maintenance of stable emulsions are crucial for product quality. The emulsion formed with *γ*-PGA and olive oil in our study is particularly noteworthy as the ES was above 50% even after 24 h of storage, and the distribution of small oil droplets was scattered with negligible droplet flocculation (Willumsen and Karlson, 1996; Linke et al., 2020). In agreement with these findings, Li et al. (2023) also demonstrated that *γ*-PGA forms complexes with soybean protein isolate (SPI), which facilitates the establishment of a stable and effective food emulsion system.

**Table 3:** Emulsifying activity and stability (24 h) of M-E6 *γ*-PGA.

Oil	EA (%)	ES (%)
Olive oil	79.33 ± 1.15^a^	70.00 ± 0^a^
Sunflower oil	44.84 ± 1.04^b^	40.00 ± 0^b^
Coconut oil	45.45 ± 0^b^	34.00 ± 0^c^

The values are expressed as mean ± SD (*n* = 3). *Parameters having superscript a and b letters denote the statistically significant difference at *p* < 0.05. EA-Emulsifying activity and ES-Emulsifying stability.

#### 3.11.5 Flocculation activity

The flocculating activity of *γ*-PGA was 29.32%, being an anionic biopolymer that imparts a negative charge capable of binding to cations and positively charged metals, resulting in bridge formation responsible for flocculation. This way, *γ*-PGA aids in flocculation by charge neutralization and bridging processes (Carvajal-Zarrabal et al., 2012; Fernandes et al., 2023). Fernandes et al. (2023) also reported an exceptionally high flocculation activity of 92% at 5 mg/L, utilizing *γ*-PGA isolated from commercial nattos comprising soy and *B. subtilis.*

### 3.12 Functional properties of γ-PGA

#### 3.12.1 Antioxidant activity

Antioxidant activity was assessed using four different assays, wherein the *γ*-PGA showed significant antioxidant activity which increased with increasing concentration (Table 4). The *γ*-PGA exhibited maximum ABTS^+^ radical scavenging of 85.52 ± 0.73% at 1 mg/mL. The transfer of hydrogen from the hydroxyl groups of *γ*-PGA effectively reduces and neutralizes ABTS^+^ free radicals (Darwish et al., 2024). The reducing activity of *γ*-PGA was measured and expressed as ascorbic acid equivalent (AAE/mg). The reducing activity was at different levels of *γ*-PGA concentration and was shown to have a good reducing activity of 155.41 ± 4.82 AAE/mg at 20 mg/mL concentration of *γ*-PGA. Hydroxy-radical scavenging activity was shown to be increased in a concentration-dependent manner, highest as 254.10 ± 5.357% at 20 mg/mL of *γ*-PGA concentration. In contrast, a lower hydroxyl scavenging activity (85.2%) was observed in a study reported at 1 mg/mL concentration (Lee et al., 2020). The FRAP assay was determined to evaluate the chelation ability of *γ*-PGA, showed a linear increase in the reduction of ferric ions, showing the highest activity at 20 mg/mL. The results align with the study performed by Quach et al. (2022) on *γ*-PGA isolated by *B. velezensis* VCN56. At a concentration of 4 mg/mL *γ*-PGA, hydroxyl scavenging activity was determined as 81% while the reducing power (FRAP) reduced to 0.6 μm Fe(II)/mL. Similarly, Galanty et al. (2025) represent improved antioxidant activity in kale sprouts supplemented with 0.01% *γ*-PGA. All the above antioxidant assays showed that this *γ*-PGA is an extremely good antioxidant agent and has the potential to be used as a natural antioxidant in food and pharma applications (Lee et al., 2014a, 2014b; Lee et al., 2020).

**Table 4:** Antioxidant activity of *γ*-PGA.

Concentration (mg/mL)	ABTS (%)	Reducing (μg/mL of AAE)	Hydroxyl radical (%)	FRAP (μm Fe(II)/mL)
0.25	39.37 ± 1.98^d^	20.37 ± 0.502^g^	21.01 ± 0.724^e^	0.62 ± 0.030^g^
0.5	62.36 ± 0.73^c^	33.34 ± 2.461^f^	56.03 ± 2.544^de^	0.69 ± 0.002^f^
1	85.52 ± 0.73^a^	63.40 ± 2.080^e^	69.32 ± 2.928^cd^	0.84 ± 0.006^e^
2.5	85.18 ± 0.44^a^	96.78 ± 2.630^d^	94.20 ± 1.917^cd^	1.02 ± 0.007^d^
5	82.12 ± 0.73^a^	124.71 ± 3.6713^c^	109.42 ± 0.724^bc^	1.36 ± 0.009^c^
10	74.96 ± 0.44^b^	136.96 ± 5.767^b^	137.68 ± 6.440^b^	2.04 ± 0.046^b^
20	73.84 ± 0.43b	155.41 ± 4.825^a^	254.10 ± 5.357^a^	3.22 ± 0.019^a^

Results are expressed as mean± standard deviation of triplicate analysis (*n* = 3). *Parameters having superscript a to g letters denote the statistically significant difference at *p* < 0.05. ABTS-2,2′-azino-bis (3-ethylbenzothiazoline-6-sulfonic acid); AAE-Ascorbic acid equivalent; FRAP-Ferric reducing antioxidant power.

### 3.13 *γ*-PGA as cryoprotectant

#### 3.13.1 Standard plate count

The cryoprotectant activity of *γ*-PGA with all the probiotic bacteria tested is shown in Table 5. Two concentrations of *γ*-PGA (5 and 10%) along with two standard cryoprotectants (10% sucrose and 10% glycerol) were used to evaluate the ability to protect the bacteria for four freeze–thaw cycles followed by storing the samples at −80°C for 24 h. Bacteria without cryoprotectant (only sterile distilled water) was treated under same conditions as control. In case of *Lmb. fermentum*, the highest loss in cell-viability, i.e., 1.22 log CFU/mL, was observed in control. *γ-*PGA at 10% demonstrated loss in cell viability by 0.02 log CFU/mL, slightly comparable to sucrose and glycerol. For *LcS. rhamnosus*, the control samples showed the greatest reduction of 1.99 log CFU/mL in cell viability. However, treatment with 5% *γ*-PGA was very effective, reducing the loss by only 0.01 log CFU/mL, surpassing the efficacy of 10% sucrose (0.04 log CFU/mL). In case of *S. thermophilus*, control showed the maximum loss, i.e., 1.27 log CFU/mL. Notably, when treated with 10% *γ-*PGA, the strain showed the least reduction (also among all the probiotics used) of 0.01 log CFU/mL. Comparatively, the reduction in cell viability exhibited was of 0.04, 0.14, and 0.12 log CFU/mL when treated with 5% *γ-*PGA, 10% sucrose, and 10% glycerol, respectively.

**Table 5:** Cryoprotectant ability of *γ*-PGA with probiotic bacteria based on standard plate count.

Cryoprotectants used	*Lmb. fermentum* (log CFU/mL)	Reduction in cell viability of *Lmb. fermentum* (log CFU/mL)	*Lcs. rhamnosus* (log CFU/mL)	Reduction in cell viability of *Lcs. rhamnosus* (log CFU/mL)	*S. thermophilus* (log CFU/mL)	Reduction in cell viability of *S. thermophilus* (log CFU/mL)
Before (F-T)-Control	7.164 ± 0.02^b^		7.396 ± 0.07^b^		6.877 ± 0.04^b^	
After (F-T)-Control	5.935 ± 0.25^a^	1.229	5.401 ± 0.45^a^	1.995	5.602 ± 0.3^a^	1.275
Before (F-T) 5%-*γ-*PGA	7.171 ± 0.1^a^		7.378 ± 0.04^a^		6.846 ± 0.02^a^	
After (F-T) 5%-γ-PGA	7.132 ± 0.02^a^	0.039	7.359 ± 0.04^a^	0.019	6.803 ± 0.03^a^	0.043
Before (F-T) 10%-γ-PGA	7.208 ± 0.01^a^		7.415 ± 0.05^a^		6.834 ± 0.05^a^	
After (F-T) 10%-γ-PGA	7.178 ± 0.02^a^	0.029	7.380 ± 0.06^a^	0.035	6.819 ± 0.05^a^	0.015
Before (F-T) 10%-Sucrose	7.171 ± 0.01^a^		7.446 ± 0.03^a^		6.785 ± 0.05^a^	
After (F-T) 10%-Sucrose	7.146 ± 0.00^a^	0.025	7.403 ± 0.02^a^	0.043	6.734 ± 0.05^a^	0.142
Before (F-T)10%-Glycerol	7.126 ± 0.01a		7.39 ± 0.01^a^		6.876 ± 0.02^a^	
After (F-T)10%-Glycerol	7.096 ± 0.03a	0.029	7.364 ± 0.07^a^	0.026	6.755 ± 0.06^a^	0.121

Results are expressed as mean± standard deviation of hextuplet analysis (n = 6). Values marked with letter a and b are significantly different (*P* < 0.05). F-T: Freeze – Thawing.

Cryoprotectant activity of *γ*-PGA was better than sucrose and glycerol (*p* < 0.05) with *LcS. rhamnosus* at 5% *γ*-PGA, in addition, *S. thermophilus* showed effective results at 5 and 10% *γ*-PGA both. *γ*-PGA demonstrates effective activity in protecting probiotic bacteria throughout freeze–thawing cycles at concentrations of both 5 and 10% *γ*-PGA when compared to the negative control (probiotic without cryoprotectant). The study concludes no significant difference between before and after freeze-thawing when *γ*-PGA was used as cryoprotectant (*p* > 0.05), whereas in the control without any cryoprotectant, there was significant loss of viable cells (p < 0.05). The cryoprotective activity of *γ*-PGA has been previously assessed (Mitsuiki et al., 1998; Bhat et al., 2013). Similar outcomes have been reported by Bhat et al. (2013) with *Lb. paracasei* at 10% *γ*-PGA, whereas with 5% *γ*-PGA, the activity found was less than the standard cryoprotectant used in their study (10% sucrose). In contrast, our study showed enhanced cryoprotectant activity at a lower concentration of 5% *γ*-PGA, making it a superior candidate in the field. The freeze-dried encapsulated *S. thermophilus* 937 shown to have a 90.59% viable cell count when encapsulated with a combination of cryoprotectants consisting of sucrose/ skim milk/ sodium alginate, the result aligns with our study, marking better activity with only *γ*-PGA (Di et al., 2023).

Generally, *γ*-PGA is considered a good cryoprotectant mainly through its molecular structure rich in carboxyl groups that enable the formation of hydrogen bonds with water molecules. The process inhibits ice crystal formation and recrystallization as a result of strengthening with bacterial proteins through its hydrogen bonds. *γ*-PGA make strong hydrogen bond with water molecules absorbing onto the surface of ice thereby restricting water movement during freezing. Also, the carboxyl groups on *γ*-PGA can form protective electrostatic interactions with the lipid bilayers of cells, especially those of sensitive probiotics and proteins (Parati et al., 2023). Additionally, *γ*-PGA can increase solution viscosity forming a protective matrix that traps water thus preventing intracellular osmotic shock (Liu et al., 2025). All these functionalities set it apart from traditional commercial cryoprotectant agents like sucrose, sorbitol, trehalose, hydroxyethyl starch, and polyphosphates. In addition, osmotic pressure of these compounds also play a crucial role permeating through the cell walls. Sucrose, permeates easily while polyglutamic acid, being strongly hydrophilic does not cross membranes, retain water externally. Glycerol initially draws water out gradually entering into the cells, reducing osmotic gradients over time (Roy et al., 2016; Ko and Gross, 1998). These versatile properties makes it a valuable candidate in applications ranging from bacterial, mammalian cell preservation to food and pharmaceutical industries (Stolzing et al., 2012; Bhat et al., 2013; Zhang et al., 2021; Liu et al., 2025; Manika et al., 2025).

#### 3.13.2 Flow cytometry

The fluorescent propidium iodide (PI) stain distinguishes the dead cells/viable but non-culturable cells (VBNC) from live cells in a mixed population by staining only dead cell nuclear DNA content with damaged cell membranes (Pazos-Rojas et al., 2023; Rosenberg et al., 2019). VBNC is the mechanism of bacteria to endure harsh environments such as temperature change, nutrient starvation, or osmotic stress at which they can potentially enter a culturable state under optimum conditions, but lose the ability to grow on conventional media (Oliver James, 2005).

**Figure 8:**
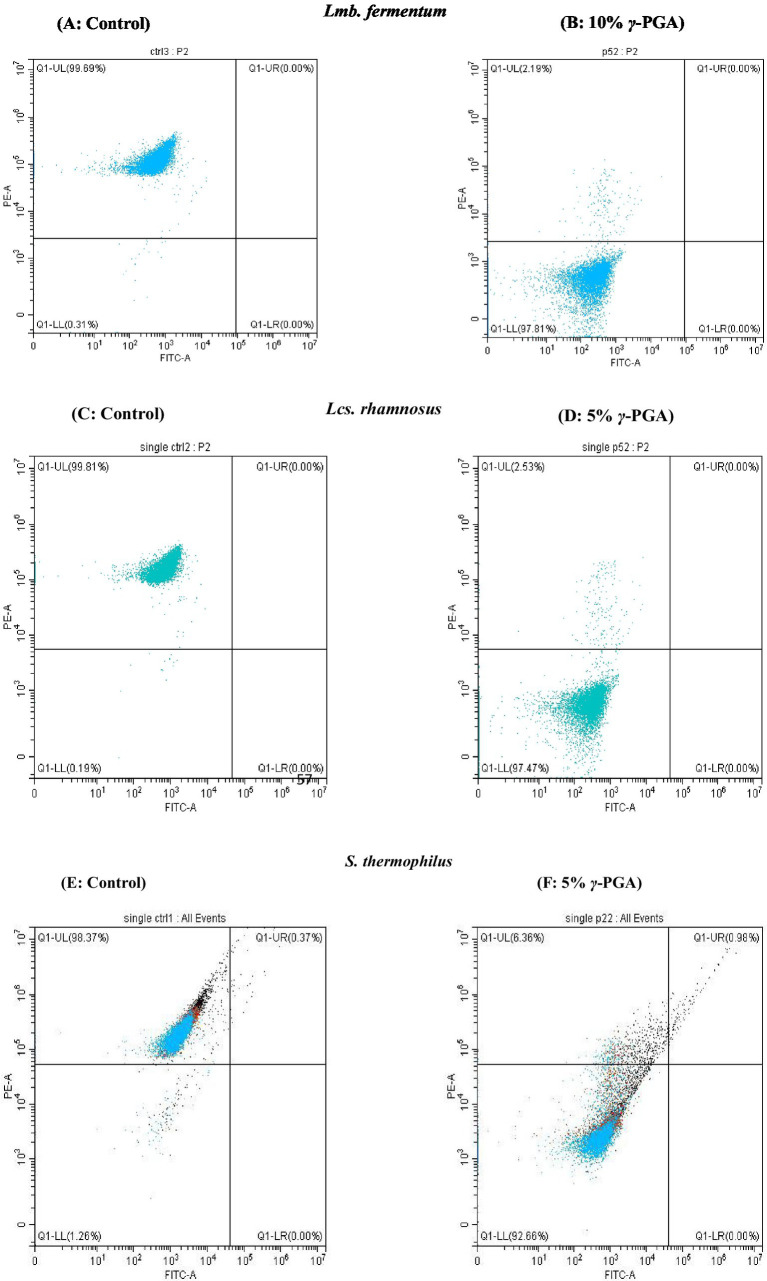
Flow cytometry analysis showing the cells staining **(A,B)** *Limosilactobacillus fermentum*- control and *γ*-PGA -10% **(C,D)** *Lacticaseibacillus rhamnosus*: control and *γ*-PGA -10% **(E,F)** *S. thermophilus*-control and *γ*-PGA − 5% as cryoprotectant.

The results acquired by flow-cytometry revealed that the cell population was segregated into two quadrants upon single fluorescence staining with PI, as illustrated in Figure 8: PI negative/ live population on the left lower (Q1-LL) and PI positive/dead population on the upper left (Q1-UL) were observed. The effect of *γ*-PGA as a cryoprotectant on three different probiotic strains were compared with sucrose and glycerol. The Flow Cytometry plots in Figure 8 depicts *γ*-PGA treated probiotic strains with their corresponding unstained control. On probiotic strain *Lmb. fermentum*, the effect of 5% *γ*-PGA on the viable cell percentage (84.53%) after freeze-thaw cycle was almost similar to sucrose (89.96%), and glycerol (91.55%). However, with 10% *γ*-PGA, the percentage of live cells increased to 97.51%, which was considerably more than both sucrose and glycerol as shown in Figures 8A,B (Stained *Lmb. Fermentum*). In the case of *LcS. rhamnosus,* this was 96.08 and 97.57% with 5 and 10%-*γ*-PGA, respectively. The percentage of viable cells was equivalent to sucrose (96.77%) and significantly higher than glucose (91.09%) (Figures 8C,D). In addition, *S. thermophilus* showed the maximum cell viability (93.47%) at 5% *γ*-PGA compared to any other cryoprotectants used (Figures 8E,F). Therefore, by flow-cytometry analysis, the live and dead bacterial population count was confirmed quickly with more accuracy compared to the plate-count method. However, the cell viability (CFU/mL) obtained through the plate count method showed lower counts compared to those detected by flow cytometry. The reason for this difference may be the metabolically active and viable fraction of probiotic cells, entered a VBNC state, thereby failing to form colonies and did not account for viability in the traditional plating method. In contrast, in advanced flow-cytometry techniques, a direct rapid enumeration of viable probiotic cells via nuclear staining was possible, thus enumerating viable cells more accurately (Ananta et al., 2005; Davis, 2014; Deza et al., 2017). Both traditional methods of bacterial viable cell count through the plating method, as well as the rapid and reliable method of flow cytometry enumeration, showed similar trends.

## 4 Recent advances in *γ*-PGA applications and limitations

Recent research has significantly advanced the applications of *γ*-PGA, demonstrating its versatility across various applications. In biomedicine, *γ*-PGA based nano-carriers have propelled cancer therapy by enabling targeted drug delivery for chemotherapeutic drugs like paclitaxel and doxorubicin (Rizwan et al., 2025). The production microgels utilizing *γ*-PGA isolated from *Bacillus* spp. is one of the recent innovations having applications in multiple fields like agriculture, medicine and environmental management (Pal et al., 2024). Recently, *γ*-PGA has been studied for foliar application to enhance the yield of various crops; maize yield has been reported to be increased by 37% (Wang et al., 2025).

While *γ*-PGA possesses remarkable applications across diverse sectors, including food, pharmaceuticals, healthcare, and agriculture, some limitations hinder its widespread adoption and commercial viability (Elbanna et al., 2024). The presence of different enantiomers due to its chiral diversity and microbial origin influences biocompatibility and toxicity profiles posing regulatory challenges. Additionally, stability under physiological conditions and consistent quality are demanding, presenting hurdles for clinical use. A significant challenge lies in the scalability of *γ*-PGA production, where current methods often struggle to meet the demands for large-scale industrial applications. Optimization of fermentation processes, strain selection and medium composition remains a challenge for production at industrial scale (Elbanna et al., 2024). On the other hand, production of *γ*-PGA is expensive, attributing to its low yield and productivity in industrial processes, necessitating process optimization and genetic engineering to reduce costs and enhance yield. The efficiency of these processes can vary significantly depending on the specific production strain, fermentation conditions, and purification methods utilized, leading to inconsistencies in product quality and increased manufacturing costs (Nair et al., 2023). The regulatory compliance, high production costs, and limited scalability remain substantial barriers for the broader adoption of polyglutamic acid in industry.

## 5 Conclusion

In this study, the physicochemical and functional properties of *γ*-PGA isolated from *Bacillus* sp. M-E6 of fermented food origin were described. The *γ*-PGA displayed a considerably high decomposition temperature range, making it suitable for incorporation into baking applications to enhance texture. In addition, *γ*-PGA exhibited significant water solubility and water-holding capacity. The porous nature of *γ*-PGA, as shown by SEM, renders it suitable for various applications in the food industry, serving as a stabilizer and thickening agent, while also being conducive for the preparation of hydrogels. The *γ*-PGA showed significant cryoprotective properties and significant *in vitro* antioxidant activity. These functional characteristics suggest its potential as a biopolymer with diverse applications. Its wider functional roles, particularly in improving food texture and emulsification stability and acting as an efficient cryoprotectant in probiotic-based frozen food compositions, require thorough investigation and validation. Furthermore, due to its biocompatibility, biodegradability, and functional diversity, *γ*-PGA has potential applications in a wide range of industries, including food, pharmaceuticals, and medicine. This study contributes significantly to the advancement in *γ*-PGA research by demonstrating unparalleled functional properties of *γ*-PGA, and it is distinctly different from previous work that primarily evaluated *γ*-PGA only in composite systems.

## Data Availability

The datasets presented in this study are publicly available. This data can be found at: https://www.ncbi.nlm.nih.gov/genbank, accession number PV555073.
