# Supplementary material for: Microbial production and functional assessment of γ-polyglutamic acid isolated from Bacillus sp. M-E6
Source: Front Microbiol. 2025 Oct 15;16:1647287. doi: 10.3389/fmicb.2025.1647287 (PMC12568500; doi:10.3389/fmicb.2025.1647287)
Supplement: Supplementary file 1 [file Data_Sheet_1.docx]

**Microbial production and functional assessment of *γ*-polyglutamic acid isolated from *Bacillus* sp. M-E6**

Verma Manika^a^, Palanisamy Bruntha Devi^a^, Jessica Majaw^a^, Potunuru Uma Rani ^a^, G. Bhanuprakash Reddy ^b,^ Digambar Kavitake ^a,b,^*, Prathapkumar Halady Shetty ^a,^*

***Supplementary Data***

**Figure_S1 (A&B)**


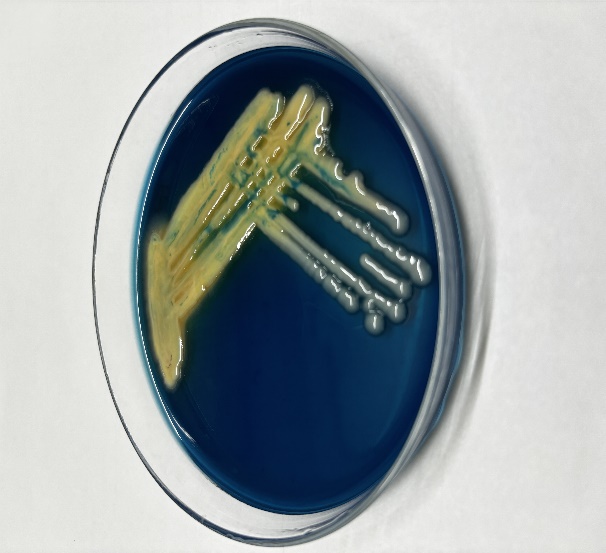

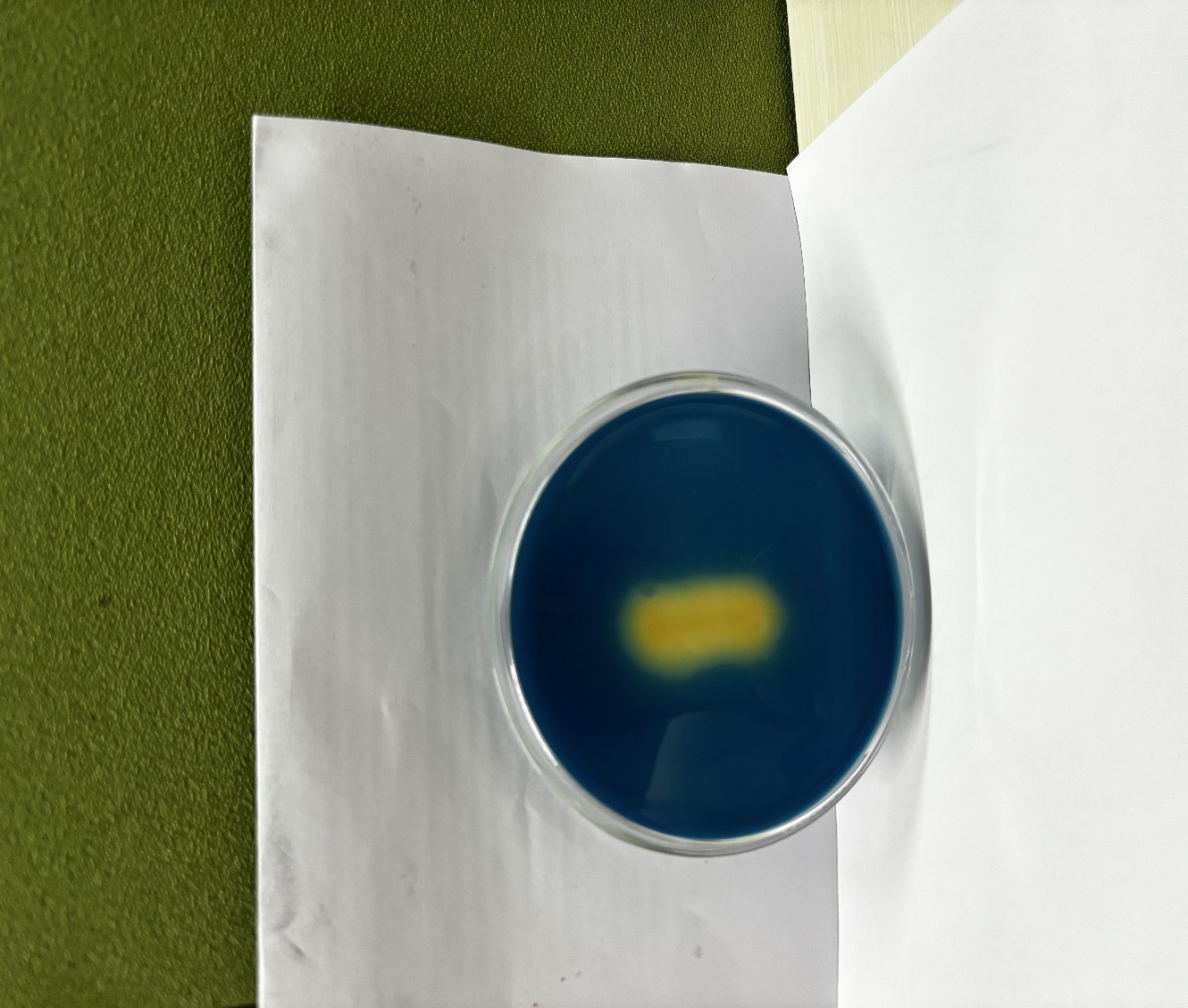


(a)

(b)

**Figure_S1:** Screening of potential *γ*-PGA producers on production medium containing methylene blue dye (A) with a prominent mucous layer (B) showing characteristic concentric zone around the *Bacillus* sp. M-E6 colony patch

**Figure_S2 (A&B)**


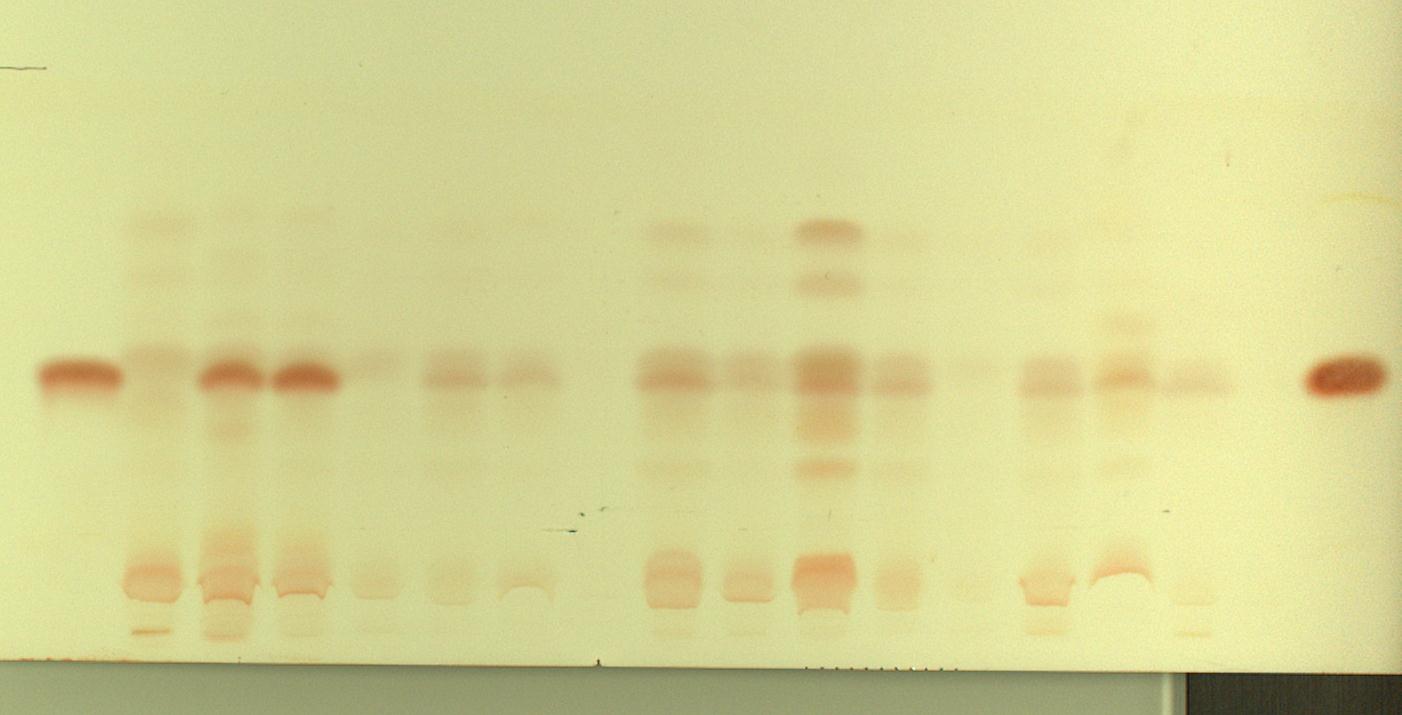


L-glutamic acid

M-E6

M-4

M-J7

M-SWR

C5

Ibj6

Y3

HG1

HG2G2

GG1

GG2

E2

E3

F1

G3

G4

(a)


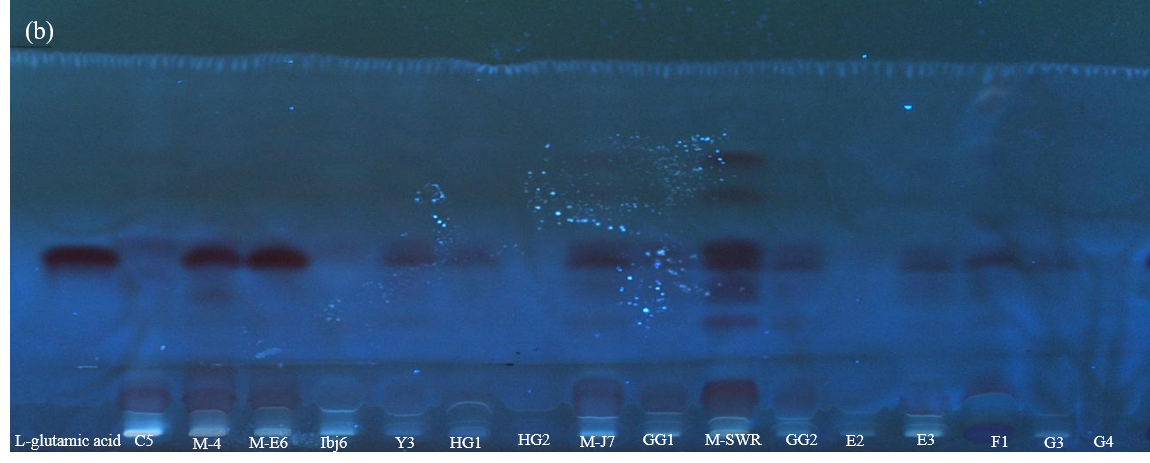


**Figure_S2:** Chromatogram showing hydrolyzed *γ*-PGA (glutamic acid units) produced by the 16 isolates corresponding to standard glutamic acid (A) under white light (B) under UV light

**Figure_S3**

**
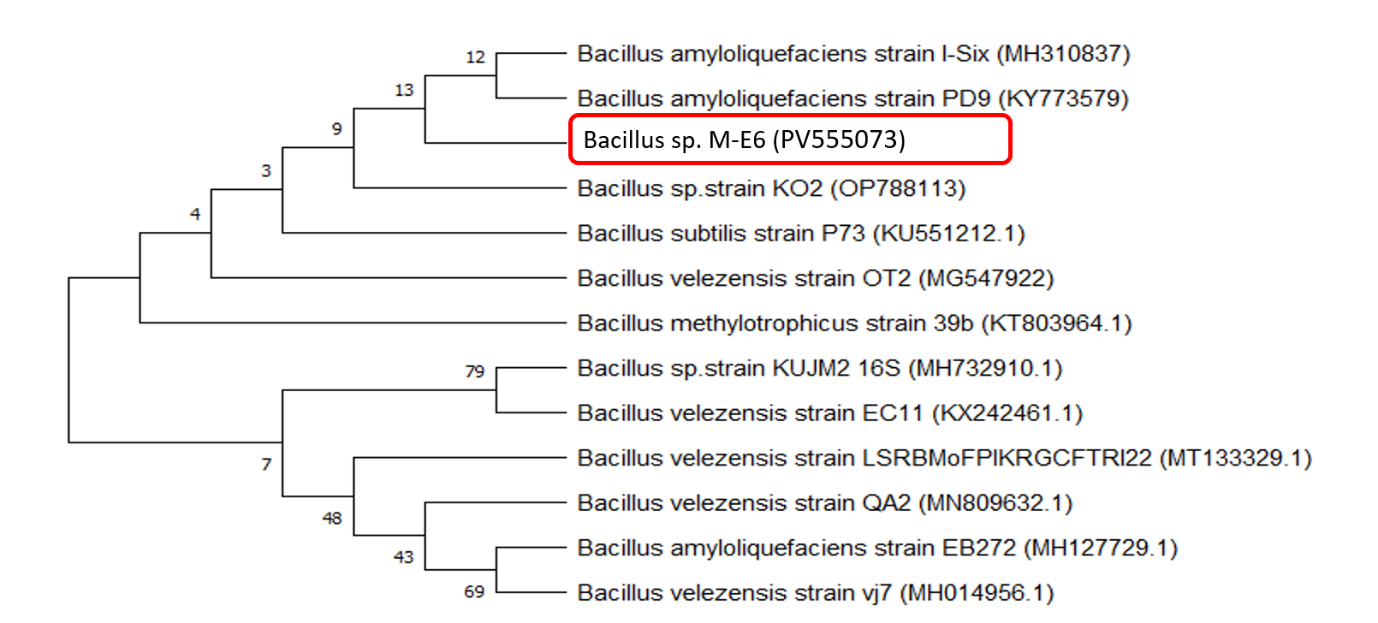
**

**Figure_S3:** Phylogenetic analysis of 16S rRNA sequences of M-E6 showing 99% similarity to *Bacillus* sp. M-E6 (PV555073) and related taxa.

**Figure_S4**


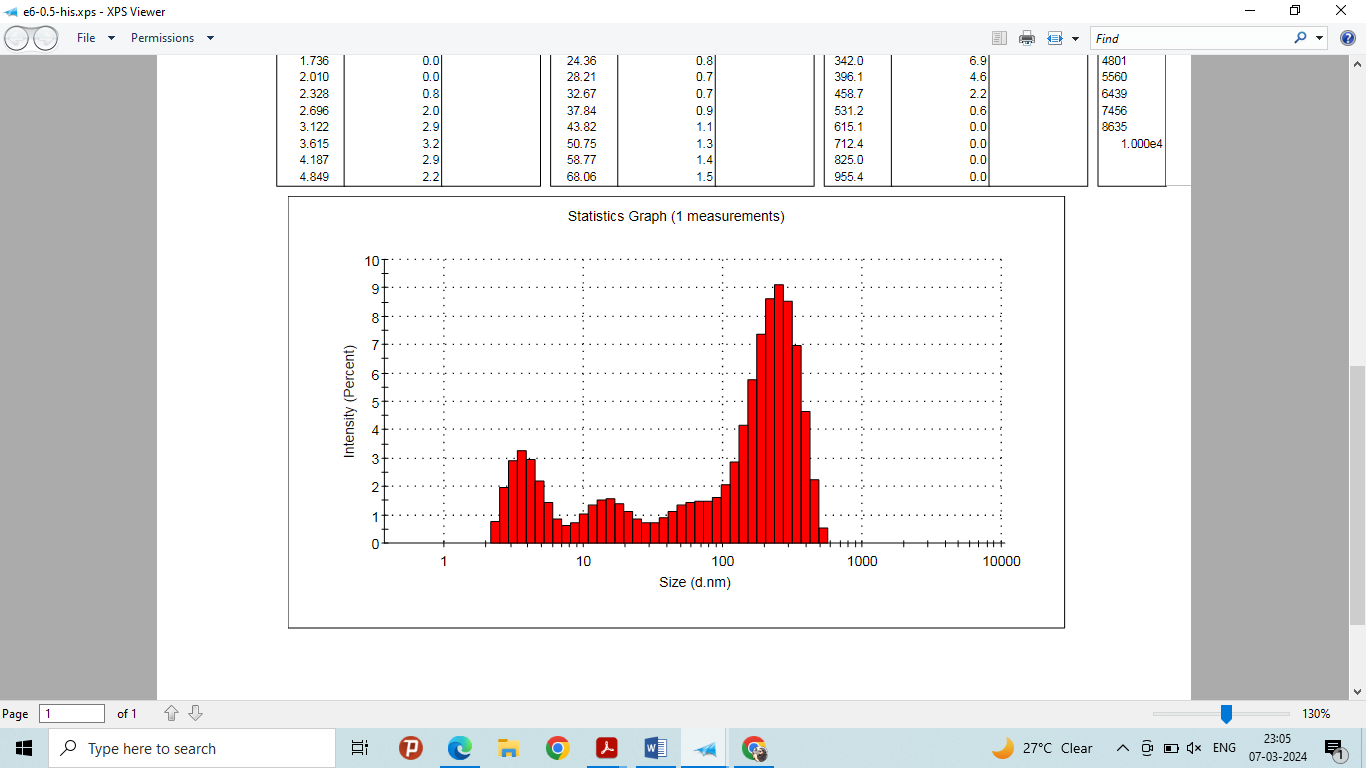


**Supplementary_S4:** Particle size analysis of *γ*-PGA measured using dynamic light scattering (DLS) method
